# Predicting NOx Distribution in a Micro Rich–Quench–Lean Combustor Using a Variational Autoencoder

**DOI:** 10.3390/e25040604

**Published:** 2023-04-01

**Authors:** Peiliang Yan, Weijun Fan, Rongchun Zhang

**Affiliations:** 1 School of Energy and Power Engineering, Beihang University, Beijing 100191, China; 2 Research Institute of Aero-Engine, Beihang University, Beijing 100191, China

**Keywords:** NOx, low-heat-value gas, RQL combustor, variational autoencoder

## Abstract

Micro gas turbines are widely used in distributed power generation systems. However, the combustion of gas turbine combustors produces a large amount of nitrogen oxides (NOx), which pollute the environment and endanger human life. To reduce environmental pollution, low-emission combustors have been developed. In recent years, there has been an increasing focus on the use of low-heat-value gas fuels, and it is necessary to study the NOx emissions from low heat value gas fuel combustors. Data-driven deep learning methods have been used in many fields in recent years. In this study, a variational autoencoder was introduced for the prediction of NOx production inside the combustor. The combustor used was a micro rich–quench–lean combustor designed by the research group using coal bed gas as a fuel. The internal NO distribution contour was obtained as the dataset using simulation methods, with a size of 60 images. The model architecture parameters were obtained through hyperparameter exploration using the grid search method. The model accurately predicted the distribution of NO inside the combustor. The method can be applied in the prediction of a wider range of parameters and offers a new way of designing combustors for the power industry.

## 1. Introduction

Distributed power generation is widely recognized as a practical solution to complement centralized power generation. It provides a flexible mode of power generation that can minimize the losses incurred during long-distance transmission. Micro gas turbines are an integral component of distributed power generation systems and have been extensively adopted worldwide [1]. However, the combustion of fossil fuels in micro gas turbines produces substantial nitrogen oxide (NOx) emissions, which have contributed to environmental degradation since the onset of industrialization [2,3,4]. Excessive NOx emissions not only pose a threat to human health by causing respiratory disease, but also damage vegetation and buildings by triggering the formation of acid rain [5]. Nitrogen oxides are also involved in ozone depleting chain reactions in the stratosphere; accordingly, an increased emission of NOx contributes to ozone layer degradation. It is now widely accepted that controlling NOx emissions is imperative. Therefore, it is of utmost importance to control NOx emissions from micro gas turbines in the field of distributed power generation.

Historically, combustible gases used for industrial purposes were preferred based on their high heat value due to their effortless combustibility and high combustion stability [6,7,8]. Natural gas, containing less than 2% nitrogen in practical industrial applications, is a crucial high-heat-value gas for human consumption. Conversely, natural gas with over 6% nitrogen is deemed to be of low heat value and must undergo a nitrogen removal process [9]. Despite the existence of vast reserves of extractable combustible gases around the world, some of them, such as coal bed methane and landfill gas, are low-heat-value gases. In developing countries, significant volumes of low-heat-value gases were previously squandered for various reasons. In recent years, there has been a gradual shift in societal attitudes toward increased environmental protection, and low-heat-value gases are now being valued as alternative fuels [10,11,12,13,14]. Instead of being treated as waste gases, their unique characteristics make them ideal for use as fuel. However, the combustion of low-heat-value gases in turbine engines results in different NOx emissions to those of conventional fuels. As a result, there has been increased research into gas turbine combustors that utilize low-heat-value gas fuels.

Reducing pollution emissions from gas turbine units is heavily dependent on controlling the combustion process in the gas turbine combustor. To this end, research is being conducted on low-pollution combustor structures, such as the rich-burn quick-quench lean-burn (RQL) combustor [15], which is favored in industry due to its simplicity. Heavy-duty combustion turbines typically incorporate this combustor in practical applications [16]. However, micro gas turbines, despite their higher pollution emissions, including NOx, compared to their heavy-duty counterparts, have a lower overall efficiency. Additionally, the combustion characteristics of low-heat-value gas fuels necessitate an investigation into the combustion generation patterns of these gases inside the micro RQL combustor.

NOx production in a gas turbine combustor is influenced by various parameters, including jet momentum flux [17], combustion equivalence ratio [18], flame temperature [19], total pressure loss [20], high-temperature zone residence time [21], airflow velocity [22], fuel composition [23], fuel–air mixture homogeneity [24], etc. Due to the numerous factors that influence NOx production, researchers have conducted a considerable number of experimental tests on the RQL combustor. Meisl et al. discovered that increasing pressure affected the mixing of the RQL gas stream and resulted in changes in NOx emissions [25]. Holdeman et al. found that varying the number of orifices and inlet air temperature could also alter NOx emissions [26]. Göke et al. investigated the effect of inlet air humidity on NOx generation [27], while Laranci et al. explored the impact of the combustor’s length on NOx production [28]. Oechsle et al. examined the effect of the number of quench holes on the reaction in the RQL combustor [29], and Li et al. investigated the influence of quench hole arrangement on NOx emissions from the RQL combustor [20].

One of the primary objectives when designing industrial combustors is to minimize NOx production within the combustor. Achieving this objective requires a deep understanding of NOx distribution within the combustion field under varying operating conditions, which is critical when developing gas turbine combustors. Currently, experimental and numerical simulation methods dominate the study of combustors, as previously noted [30,31,32]. While these methods have brought significant findings to various aspects of combustor research, they both possess limitations. The experimental approach necessitates the creation of multiple models, and even with an abundance of experimental data, human understanding of combustion field mechanics is inadequate, requiring new models for even minor combustor adjustments. Conversely, numerical simulation addresses the issue of generating costly experimental models with extensive experimental cycles, but requires a significant amount of simulation time. Moreover, as researchers aim for greater simulation accuracy, simulations become progressively more computationally resource intensive.

Data-driven prediction methods, commonly referred to as artificial intelligence methods, have recently surged in popularity. This approach eliminates the need for access to the combustor chemical mechanism, enabling direct prediction of the desired target using input parameters. Artificial intelligence methods offer significant advantages over traditional methods, particularly when ample data is available, as it saves valuable experimental or computational resources. The field of combustion has already shown promising research results through the application of artificial intelligence. For example, An et al. [33] leveraged a neural network as an auxiliary solver for computational fluid dynamics methods to predict the distribution of flow and temperature fields in a combustor. Zhou et al. [34] investigated the use of deep learning based on time-averaged flame images to monitor combustion instabilities. Despite these efforts, there is still limited research on applying deep learning in combustion, with no current study examining NOx generation within RQL combustors.

The variational autoencoder (VAE) [35] is an exceptional generative algorithm that has proven to be highly effective in image prediction, and has been employed by researchers to predict standard flame models. What sets it apart from other generative algorithms is its ability to leverage the auto-encoder concept, which enables learning and reconstruction mappings into data using latent vectors, thereby facilitating directed generative data. In this regard, the VAE algorithm is utilized in this study to predict the distribution of NOx in the interiors of RQL combustors fueled by low-heat-value gas.

The study’s main contribution is the introduction of the VAE method into the field of gas turbine combustor pollution emissions, specifically for predicting internal NOx generation. The method can be applied to predict flow fields and other aspects because it is a universal method.

## 2. Materials and Methods

### 2.1. Structure of the Combustor

**Figure 1:**
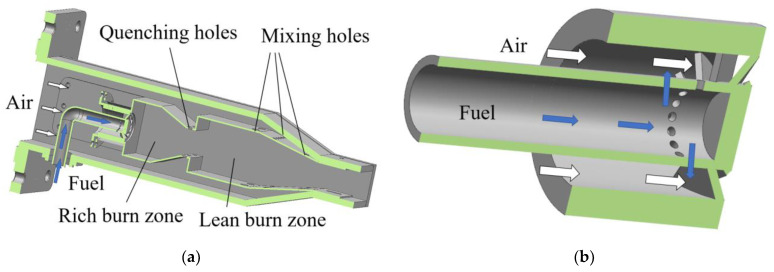

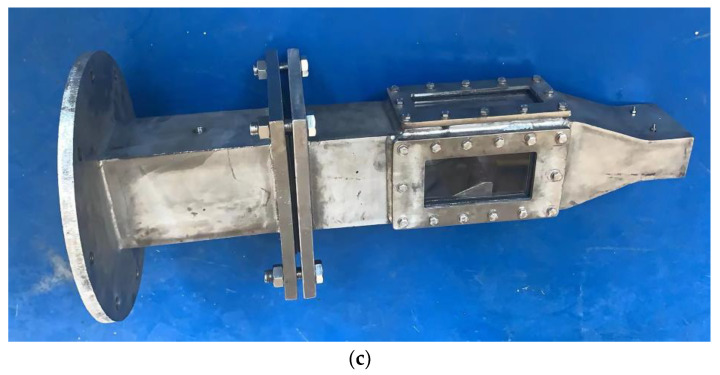
The self-designed micro RQL combustor structure. (**a**) Combustor model; (**b**) swirler model; (**c**) micro RQL combustor.

**Figure 2:**
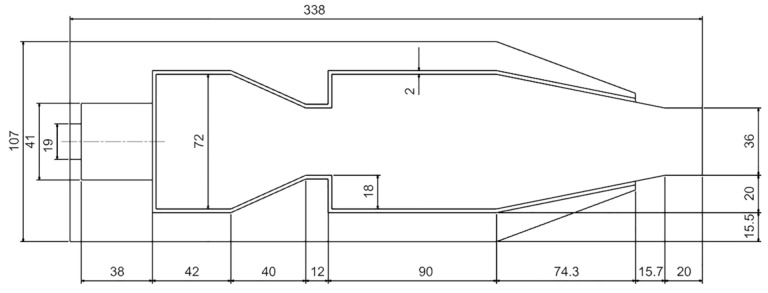
The numerical simulation model dimension.

The self-designed low-heat-value gas RQL micro combustor for this study is shown in Figure 1 [36]. The inlet air enters the combustor through the swirler, the quenching hole and the mixing hole, respectively. The direction of the air inlet to the combustor and the location of each zone are indicated in the figure. The gas in the rich-burn zone of the combustor consists only of the fuel–air mixture from the swirler, while the mixture in the lean burn zone consists of a mixture of gas from the rich-burn zone and air from the quenching hole. The swirler is designed as a partially premixed single stage swirler. The fuel gas flows out of the side orifices in front of the swirler blades and is subsequently mixed with air before passing through the blades into the combustor. The swirler structure is shown in Figure 1b, where the flow direction of the air as well as the fuel is marked. This combustor is not designed with cooling holes to ensure reliable flow distribution. The overall dimensions of the combustor are a length of 483 mm, a width of 96 mm, and a height of 123 mm. As shown in Figure 2, in order to save computational resources, the three-dimensional model used for the simulation removes part of the interfaces to the combustor. The fuel used in the combustor is coal bed methane (CBM), a low-heat-value gas that is released when mining coal. The components of CBM in this study are 30% nitrogen and 70% methane.

### 2.2. Simulation Methods and Validation

The flow inside the combustor is sophisticated, and in this study, the flow field could be described using the following control equations under the assumption that the flow field is continuously compressible and turbulent.

(1) $$\frac{\partial (\rho u\varphi )}{\partial x}+\frac{\partial (\rho v\varphi )}{\partial y}+\frac{\partial (\rho w\varphi )}{\partial z}=\frac{\partial }{\partial x}\left(\Gamma_{\phi }\frac{\partial \varphi }{\partial x}\right)+\frac{\partial }{\partial y}\left(\Gamma_{\phi }\frac{\partial \varphi }{\partial y}\right)+\frac{\partial }{\partial z}\left(\Gamma_{\phi }\frac{\partial \varphi }{\partial z}\right)+S_{\phi }$$

The expressions *φ*, *Γ_φ_*, and *S_φ_* in the different equations are shown in Table 1.

The turbulence phenomenon was described utilizing the selected shear stress transport (SST) k-ω two-equation model.

(2) $$\frac{d\rho k}{dt}=\frac{\partial }{\partial x_{j}}\left[\left(\mu +\sigma_{k}\mu_{t}\right)\frac{\partial k}{\partial x_{j}}\right]-\rho \bar{{u^{\prime }}_{i}{u^{\prime }}_{j}}\frac{\partial u_{i}}{\partial x_{j}}-\beta^{*}\rho \omega k$$

(3) $$\frac{d\rho \omega }{dt}=\frac{\partial }{\partial x_{j}}\left[\left(\mu +\sigma_{\omega }\mu_{t}\right)\frac{\partial \omega }{\partial x_{j}}\right]-\rho \bar{{u^{\prime }}_{i}{u^{\prime }}_{j}}\frac{\max\left(a_{1}\omega ;\Omega F_{2}\right)}{a_{1}k}\left(\frac{\beta }{\beta^{*}}-\sigma_{\omega }\frac{\kappa^{2}}{\sqrt{\beta^{*}}}\right)\frac{\partial u_{i}}{\partial x_{j}}-\beta \rho \omega^{2}+2\left(1-F_{1}\right)\rho \sigma_{\omega }\frac{1}{\omega }\frac{\partial k}{\partial x_{j}}\frac{\partial \omega }{\partial x_{j}}$$

The combustion phenomenon was comprehensively investigated in this study, employing the flamelet-generated manifold probability density function (FGM-PDF) model. According to the FGM model, the entire flame can be segregated into numerous, easily calculable one-dimensional flames or flamelets, which facilitates the computation of chemical reaction data prior to the flow field simulation. During simulation, critical flame state quantities can be extracted directly from the pre-calculated data. To simulate the turbulent combustion field, the PDF method was utilized as a popular solution technique in the combustion field. This probabilistic statistical approach describes the combustion process considering velocity and temperature of the turbulent flow field as independent variables. Chemical components within the flow field are computed through integration operations based on these variables. The combustion mechanism was modeled using GRI-Mech 3.0. The numerical simulation was carried out utilizing Open-FOAM V9, and the computational fluid dynamics model employed the SIMPLE pressure-velocity coupling scheme.

**Figure 3:**
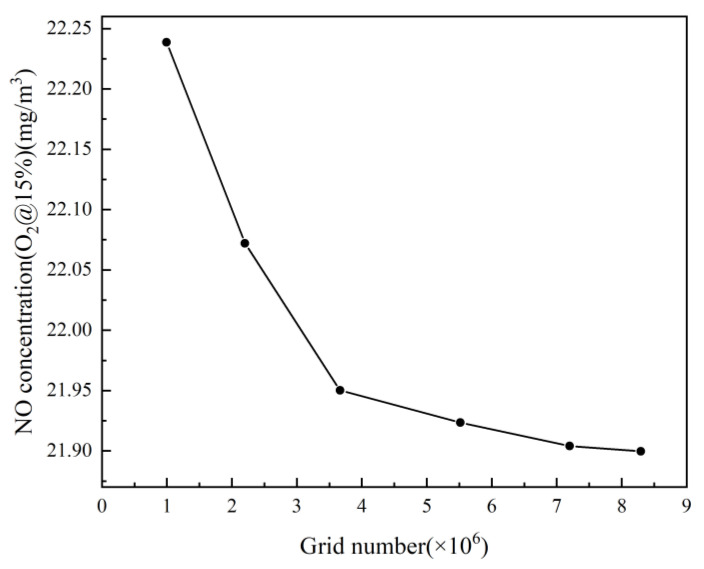
Grid independences study.

For the geometric model used in this study, six cases with different grid numbers were tested and each simulation case converged after approximately 20 h of computation. The results obtained are shown in Figure 3. After weighing the computational resources and the computational accuracy, a case with a grid number of 5.5 million was finally adopted for this study.

**Figure 4:**
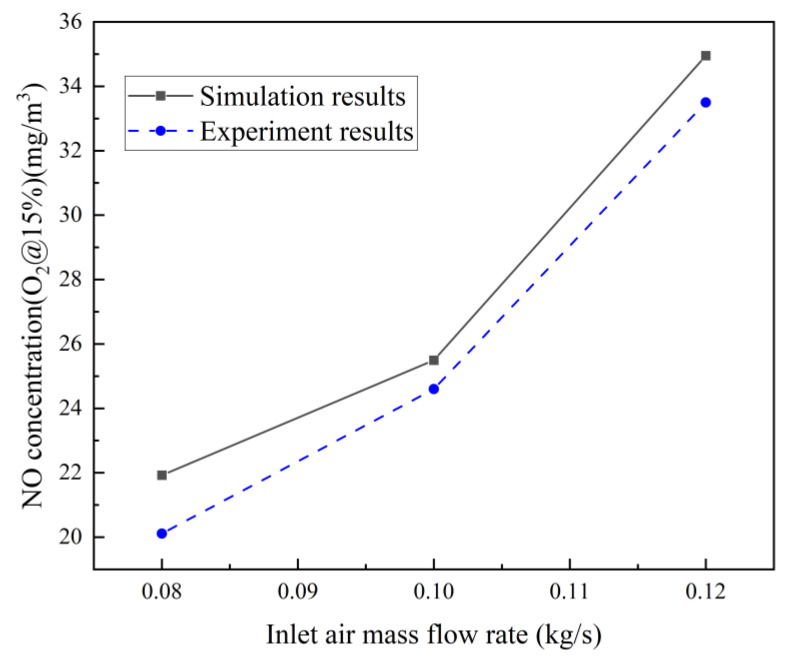
Comparison of NO concentration obtained from the present work and experiment data.

The validation of the simulation model was achieved by performing experiments on a self-designed experimental bench. To validate the numerical model in this study, identical cases to those presented in our group’s previous publication were employed [36]. The experimental and simulation cases employed the same parameters. To facilitate comparison, the NO concentrations in Figure 4 were transformed into NO concentrations at 15% oxygen content utilizing the conversion equation:

(4) $$c\left(\mathrm{NO}\right)=c^{\prime }\left(\mathrm{NO}\right)\times \frac{21-V\left(O_{2}\right)}{21-V^{\prime }\left(O_{2}\right)}$$

At the outlet of the micro RQL combustor, the concentration of NO exhaust gas is measured using a flue gas analyzer, namely, the DF-FGL flue gas analyzer manufactured by Nanjing Fangnuo Environmental Protection Equipment Company, Nanjing, China. The comparison results in Figure 4 show that the numerical model used in this study is reliable.

### 2.3. Artificial Neural Networks and Variational Auto-Encoders

An artificial neural network (ANN), first proposed in 1943 [37], is an arithmetic model, which first modelled how biological neurons work together within the biological brain. Since then, artificial neural network architectures have evolved. The creation of the error back-propagation algorithm [38] has made it possible to overcome some of the difficulties in training deep learning, and it has become practical. The introduction of convolutional neural networks greatly reduced the computational complexity of image processing, leading researchers to develop various neural networks for achieving different objectives based on this advancement.

An auto-encoder (AE) [39] is a special type of convolutional neural network that can perform data re-expression and dimensionality reduction. The network can be considered to consist of two parts: an encoder and a decoder. The encoder’s task is to map the input data to a latent space and generate a latent vector, while the decoder reconstructs the latent vector into output data. For conditionally provided auto-encoders, the output is the same as the input.

The VAE is a directed generative neural network that builds from the ideas of the AE. The main structure of the VAE, like that of the AE, consists of two networks, the encoder and the decoder. Unlike the AE, the input to the VAE is not encoded as a hidden space data point, but as a continuous probability distribution, which gives the VAE decoder the ability to obtain directed generation in resampling.

The goal of VAE is to maximize the marginal likelihood of the output of the reconstruction, which is obtained by summing up the two terms. The first of these terms is the expected value of the log marginal mean value of the data set, which indicates the error value between the generated data and the real data, and the second term is the Kullback–Leibler dispersion between the posterior distribution and the prior distribution associated with the latent variable *z*. Both distributions obey a normal distribution.

In the practical application of the algorithm, the first term, i.e., reconstruction data error, could be described using mean squared error (MSE). The second term—the Kullback–Leibler dispersion—could be calculated as

(5) $$D_{KL}\left[q\left(z|x\right)\|p\left(z\right)\right]=\frac{1}{2}\sum_{j=1}^{n}\left(\mu_{j}^{2}+\sigma_{j}^{2}-\ln\left(\sigma_{j}^{2}\right)-1\right)$$

The specific meanings of each letter of Formula (5) can be found in reference [40].

To minimize the loss function, the Adam optimization algorithm is used in order to optimize the values of each weight of the neural network.

### 2.4. Dataset

The dataset used in this study is the contour of internal NO distribution of the RQL low-heat-value gas micro combustor obtained from the simulation. The operating conditions employed are shown in Table 2. From the operating conditions shown in Table 2, 60 operating conditions were randomly selected to form a data set containing 60 images. In this study, all the data sets are based on the same legend scale, and the 20-level logarithmic coordinate scale is chosen as the legend scale due to the large difference in NO concentration between the operating conditions. All the images were converted to jpg format, the color mode was three-channel RGB mode, and the resolution of the dataset images was 377 pixels wide and 377 pixels high. The order of the images in the dataset was randomly disrupted when the dataset was created, and since the main purpose of this study is to achieve contour generation, the dataset was been processed by data enhancement.

### 2.5. Architectural Details

**Figure 5:**
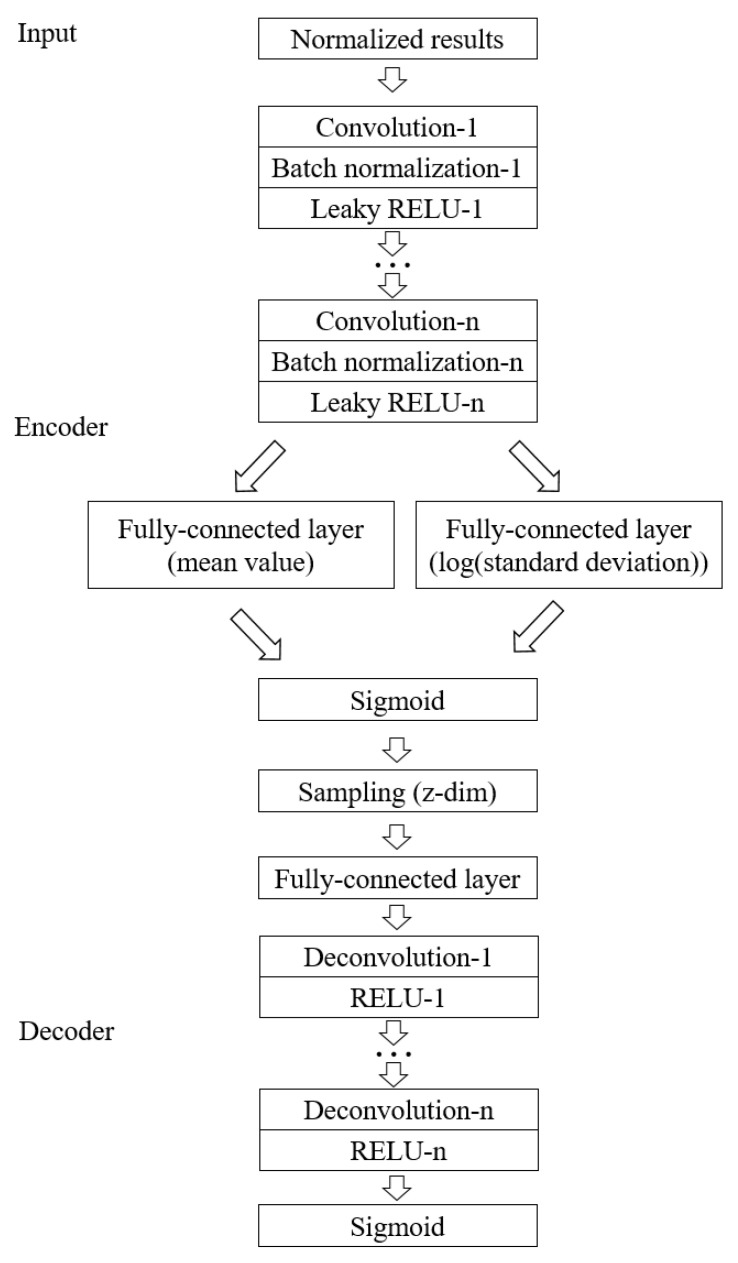
The VAE model structure used in this study.

The general architecture of the VAE model for this study is shown in Figure 5. The encoder consists of n convolutional layers and two fully connected layers, where each convolutional layer is followed by a regular layer and an activation layer, and since the original image needs to be constructed as accurately as possible, the convolutional layers are not followed by a pooling layer to reduce the error. All of the regular layers use the batch normalization layer, all of the activation layers are chosen to be processed using the Leaky RELU function, and the subsequent fully connected layers are able to obtain the mean and standard deviation for subsequent reconstruction. The final result is processed using the sigmoid function and output as a latent vector of a set dimension. The decoder is first randomly sampled and output to the fully connected layer, followed by the deconvolution layer, which has the same number of layers as the convolution layer. Similar to the convolutional layers, each deconvolutional layer is followed by an activation layer, where the RELU function is chosen for all activation functions, and a sigmoid function is applied after the last activation layer. This part of the study was implemented in Python 3.9 based on Pytorch 1.11.0.

## 3. Results

In the application of VAE models, hyperparameters can affect the performance of the prediction. These include the mini-batch size, the number of hidden layers, the kernel size, the learning rate, the latent vector dimension, the number of convolutional channels, and so on. The total number of iterations was chosen as 5000 in order to ensure that the loss function of the model cannot continue to decline, and the number of hidden convolutional layers of the encoder and decoder was chosen as three because it was found that the number of layers higher than three made the loss function significantly higher during testing. The hyperparameters were optimized using a grid search approach and the optional values of the hyperparameters are shown in Table 3.

**Figure 6:**
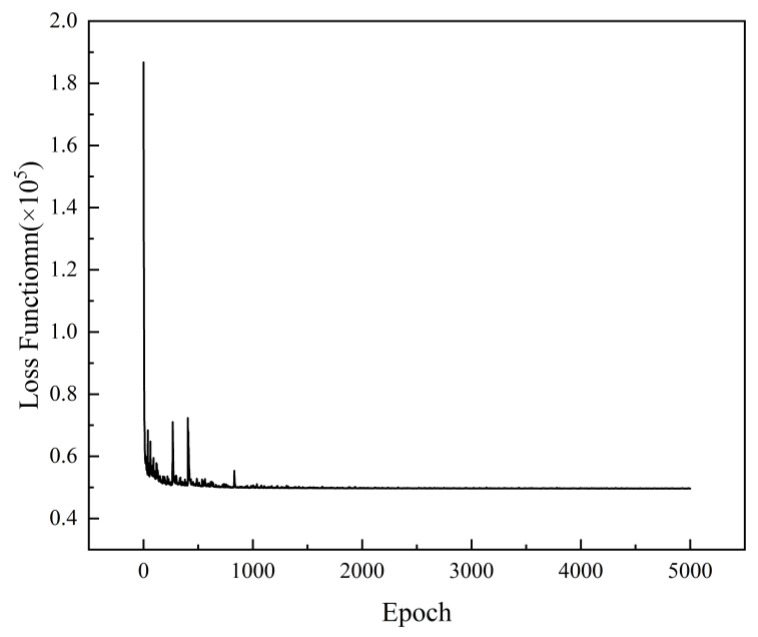
Mean loss of each image data per epoch. The result is the sum of the mean square errors of pixels in all the three channels.

Using the hyperparameters selected in the previous section, the VAE model was built and trained for 5000 epochs with a mini-batch size of 2 and an Adam optimizer. Figure 6 shows the training process of the model, where the value of the loss function drops rapidly at the beginning of the training and then converges gradually after repeated oscillations, to which the adaptive optimizer contributes significantly.

**Figure 7:**
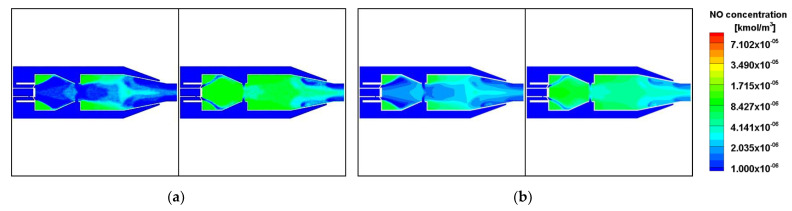
Comparison between real and generated NO distribution. (**a**) generated NO distribution (**b**) real NO distribution.

The sequential order of the training sets was randomly disordered and the results of the visual reconstruction of the two sets of data from the last mini-batch of the training set are shown in Figure 7a, while the original images are shown in Figure 7b. After decoding by the decoder, it is observed that the structure of the images is mostly accurate. Compared to the original images, the VAE reconstructed images are blurred and lose some of their detail. The blurring of the VAE generated images is mainly due to the difference between the approximate distribution and the true posterior distribution caused by the gradient noise and the VAE loss function. The errors within the flow field are mainly due to several reasons. Firstly, the data set in this study is a transient flow field obtained from simulation calculations, and the transient flow field is not averaged for each operating condition, but only the same flow time is calculated, which leads to increased inhomogeneity within the flow field. Secondly, the convolution process loses some detail, and in addition, the calculation of the main part of the loss function (MSE) is a combination of the full image results and cannot take into account the loss distribution of specific pixels within the image.

**Figure 8:**
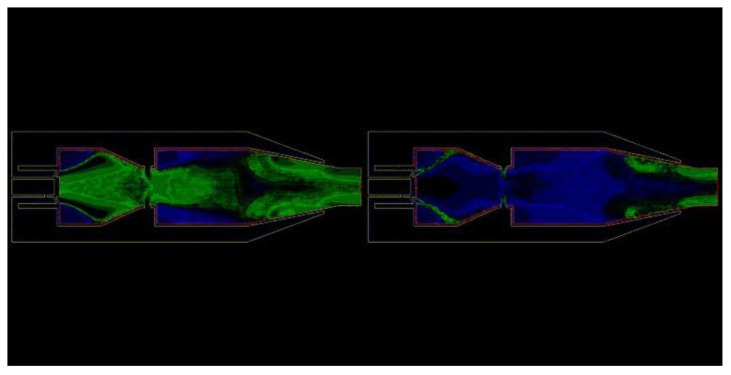
Visualizations of error in the NO distribution generated by the VAE.

Figure 8 shows the errors in the NO distribution field prediction results. It can be found that the errors mainly occur in two regions: one is the rich combustion zone and lean combustion zone of the combustor, and the other is the region from the mixing hole to the outlet of the combustor. Both of these are caused by the uncertainty of the transient simulation calculation, so the final loss cannot continue to decrease after a certain number of iteration epochs. In addition, due to the use of a logarithmic scale to generate the original contour, only a few specific colors will appear in the image, while the colors in the generated image may include color codes that do not exist in the dataset, leading to possible errors.

## 4. Conclusions

The present study presents a new data-driven technique that predicts NOx distribution inside a combustor. The proposed method utilizes a variational autoencoder to analyze the data images obtained from experiments or simulations. It is applied to anticipate the NOx distribution in a micro RQL combustor designed to operate on low-heat-value gas fuel (CBM). By generating a database of 60 images, the optimal network structure is obtained through grid search. The generator network comprises three convolutional layers and one fully connected layer. The encouraging results demonstrate an excellent agreement between the generated and actual images.

The proposed method is based on the prediction of images obtained during simulation or experiment, which can be applied in similar scenarios. For instance, particle image velocimetry results can be used to foresee the flow field inside a combustor, while planar laser-induced fluorescence results can be utilized to predict the distribution of specific flame clusters. This approach effectively reduces workload through the use of existing data and newly generated data, and offers potential for providing data for the eventual design of industrial combustors.

Nevertheless, the limited size of the dataset, comprising only 60 images of different operating conditions, represents a major hindrance for the proposed method. To increase precision, practical applications require larger datasets generated through the use of a wider range of working conditions and the averaging of results from multiple images. This would necessitate modifying the current neural network architecture, requiring additional simulation or experimental work. Finally, due to the difficulty in understanding deep learning models, adjusting hyperparameters through grid search, rather than employing specific network architecture modifications, is essential.

## Figures and Tables

**Table 1:** The meanings of the symbols in Equation (1).

Equation Type	*φ*	*Γ_φ_*	*S_φ_*
Continuity equation	1	0	*S_m_*
Momentum equation in the x-direction	*u*	*μ_e_*	*S_u_*
Momentum equation in the y-direction	*v*	*μ_e_*	*S_v_*
Momentum equation in the z-direction	*w*	*μ_e_*	*S_w_*
Energy equation	*h*	*μ_e_*/*σ_h_*	*S_h_*
Component equation	*Y*	*μ_e_*/*σ_Y_*	*S_Y_*

**Table 2:** The control parameters of the micro RQL combustor.

Parameter	Values	Unit
Inlet air temperature	400, 500, 600, 700, 800	K
Inlet air mass flow rate (corresponding lean burn zone equivalence ratio)	0.08 (0.883), 0.09 (0.783), 0.1 (0.695), 0.11 (0.623), 0.12 (0.566), 0.13 (0.518)	kg/s (None)
Swirler installation angle	40	deg
Pressure	1, 2, 3, 4	bar

**Table 3:** The alternative values and chosen values of hyperparameters.

Hyperparameter	Alternative Values	Chosen Value
Learning rate	5 × 10^−4^, 5 × 10^−5^, 5 × 10^−6^	5 × 10^−4^
Kernel size	3, 5, 7	7
Latent vector dimension	4, 16, 64	64
Convolutional channel number	8, 16, 32	32
Stride	2, 3, 5	3

## Data Availability

The research data supporting this publication are provided within the paper.

## Nomenclature

*a*_1_	model constant	*μ*	laminar viscosity [kg/(m·s)]
*c*	standard gas concentration	*μ_e_*	effective viscosity [kg/(m·s)]
*c′*	test gas concentration	*μ_t_*	turbulent viscosity [kg/(m·s)]
*F*_1_/*F*_2_	blending function	*ρ*	density [kg/m^3^]
*k*	turbulent kinetic energy [J]	*σ_h_*/*σ_k_*/*σ_Y_*/*σ_ω_*	model constants
*S_m_*/*S_u_*/*S_v_*/*S_w_*/*S_h_*/*S_Y_*	source terms	*Ω*	vorticity [1/s]
*t*	time [s]	*ω*	dissipation rate [1/s]
*u*	velocity in x direction [m/s]	Abbreviations	
*v*	velocity in y direction [m/s]	AE	auto encoder
*V*	standard gas fraction	ANN	artificial neural network
*V’*	test gas fraction	CBM	coal bed methane
*w*	velocity in z direction [m/s]	FGM	flamelet-generated manifold
Greek Symbols		MSE	mean squared error
*β*/*β**	model constants	PDF	probability density function
*κ*	model constant	VAE	variational auto-encoder
